# Time-resolved fluorescence microscopy with phasor analysis for visualizing multicomponent topical drug distribution within human skin

**DOI:** 10.1038/s41598-020-62406-z

**Published:** 2020-03-24

**Authors:** Sinyoung Jeong, Daniel A. Greenfield, Maiko Hermsmeier, Akira Yamamoto, Xin Chen, Kin F. Chan, Conor L. Evans

**Affiliations:** 1 Wellman Center for Photomedicine, Massachusetts General Hospital, Harvard Medical School, Boston, MA 02114 USA; 2 Harvard Biophysics Graduate Program, Boston, MA 02115 USA; 3 BioPharmX, Inc., 115 Nicholson Ln, San Jose, CA 95134 USA

**Keywords:** Drug delivery, Optical spectroscopy, Imaging and sensing, Acne vulgaris, Pharmacokinetics

## Abstract

Understanding a drug candidate’s pharmacokinetic (PK) parameters is a challenging but essential aspect of drug development. Investigating the penetration and distribution of a topical drug’s active pharmaceutical ingredient (API) allows for evaluating drug delivery and efficacy, which is necessary to ensure drug viability. A topical gel (BPX-05) was recently developed to treat moderate to severe acne vulgaris by directly delivering the combination of the topical antibiotic minocycline and the retinoid tazarotene to the pilosebaceous unit of the dermis. In order to evaluate the uptake of APIs within human facial skin and confirm accurate drug delivery, a selective visualization method to monitor and quantify local drug distributions within the skin was developed. This approach uses fluorescence lifetime imaging microscopy (FLIM) paired with a multicomponent phasor analysis algorithm to visualize drug localization. As minocycline and tazarotene have distinct fluorescence lifetimes from the lifetime of the skin’s autofluorescence, these two APIs are viable targets for distinct visualization via FLIM. Here, we demonstrate that the analysis of the resulting FLIM output can be used to determine local distributions of minocycline and tazarotene within the skin. This approach is generalizable and can be applied to many multicomponent fluorescence lifetime imaging targets that require cellular resolution and molecular specificity.

## Introduction

Treatments for acne vulgaris, a common skin disease affecting millions of individuals worldwide, have been developed to include a range of options, from orally administered pills to topical gels^1,2^. Recently, a topical minocycline based gel was created with the goal of achieving efficient delivery of minocycline into the skin at low topical doses^3^. Minocycline topical therapeutics may be useful for decreasing drug costs and potential side effects, such as dizziness, nausea, joint and muscle pain, and skin discoloration^4–6^. Interestingly, minocycline drugs can be made more effective when paired with retinoids^7^. Topical retinoids are also commonly prescribed for acne, and inhibit microcomedone formation, normalize keratinization and show direct anti-inflammatory activity to mitigate inflammation associated with the disease^8,9^. Therefore, combining minocycline with a retinoid in a topical formulation may provide synergistic efficacy in the treatment of acne. However, creating a multicomponent topical drug with these two active pharmaceutical ingredients (APIs) presents challenges in understanding their joint diffusion and uptake within the skin.

Determining pharmacokinetic (PK) properties of drugs is crucial for establishing proper drug dosing, regulatory standards, and usage guidelines. Topical treatments are commonly used in dermatologic and cosmetic applications, yet few methods are capable of cellular and subcellular quantification of drug penetration. Visualizing and quantifying the properties of multi-compound interactions within such formulations would allow for more accurate drug dosimetry resulting from improved understanding of where, when, and how APIs diffuse in the skin. Furthermore, understanding API PK parameters would help establish standard metrics for measuring important drug properties for desired drug targeting and activity for specific applications. Visualizing topical drug distribution in the skin can be a low-error mechanism to understand drug’s PK profile. By avoiding mathematical models of drug diffusion, and instead directly measuring drug diffusion *in situ*, researchers and physicians can develop improved drugs and formulations to achieve the proper drug-tissue interactions with reduced off-target effects.

To visualize drug uptake within the skin, several spectroscopic and optical imaging tools have been developed including mass spectroscopic imaging^10^, two-photon excitation fluorescence microscopy (TPEF)^11^, fluorescence lifetime imaging microscopy (FLIM)^12^, and autoradiographic imaging methods^13^. These tools allow for selective visualization of topical drug uptake at varying spatial resolutions. When drugs have fluorescence properties, TPEF can be a powerful tool for visualizing drug delivery. It is well suited for visualizing drug fluorescence within the skin, as it allows images to be captured deep within tissue, without significant photobleaching, photodamage, or phototoxicity associated with some single-photon excitation imaging tools. FLIM, which can be paired with TPEF, is a useful technique for situations where the endogenous skin and exogenous drug’s emission/excitation spectral fluorescent signals are similar, but fluorescence lifetimes differ. FLIM enables the visualization of individual fluorophores by using their fluorescence lifetime to provide additional molecular contrast. Recent developments in the phasor approach for FLIM analysis allow for rapid extraction of fluorescence lifetime information without the need for prior lifetime knowledge or fitting multiple exponential distributions^14–16^. The phasor analysis method transforms temporal fluorescence decay traces to a set of phasor coordinates, G and S, corresponding to the real and imaginary portions of the Fourier transformed decay traces evaluated at a specific laser repetition frequency. The transformed decay traces appear as clusters in the phasor space, where different clusters correspond to imaged fluorophores with different fluorescent lifetimes. The identity of each of these clusters can be determined by individually investigating each contributing component. For example, analyses can be carried out on images of untreated skin (to determine the endogenous autofluorescence cluster), images of the APIs themselves (to determine each exogenous drug’s cluster), and images of skin treated with individual APIs (to determine how clusters interact). Upon transformation to phasor space, the endogenous and exogenous references appear as distinct clusters, while the treated sample spans the space between those two clusters^17,18^. This is a result of phasor algebra, where multi-exponential fluorescence lifetimes are a linear combination of their individual lifetime components. This allows for computation of relative signal contribution of exogenous or endogenous references to the test sample and an accompanying visual map of signal across the tissue sample. Phasor analysis of FLIM images is especially advantageous for differentiating multiple fluorophores in heterogeneous environments, such as within the skin.

Previously, we reported using TPEF, FLIM, and phasor analysis to study the uptake of topically applied minocycline to the skin. Using FLIM and non-Euclidean phasor analysis allowed us to selectively visualize minocycline and extract its local distribution in the tissue^3,19^. In this study, we demonstrate the visualization of a multicompound drug formulation containing both minocycline and tazarotene. We quantify the local uptake of minocycline and tazarotene within the skin using a novel FLIM phasor analysis technique. The distinct detection and quantification of minocycline and tazarotene contributions, specifically the extent of compound permeation through tissue samples, is determined using a Python-based image analysis method. Compared to previously reported methods, we can now account for contributions from multiple phasor clusters (one endogenous and two exogenous references) from FLIM imaging data to quantify compound uptake in tissue. Notably, while quantification is carried out within phasor space, the results can be converted back to Cartesian space to accurately construct tissue images color coded to display local drug uptake. Compared to previous analysis methods, such as arbitrary thresholding techniques, and tedious multiexponential fitting, this newly applied approach is highly robust and yields complete drug uptake maps without missing tissue regions that otherwise would be ignored in other multicomponent phasor analysis protocols.

## Methods

### Preparation of facial skin with minocycline and retinoid treatment

Discarded human periauricular facial skin was obtained from facelift patients by BioPharmX and stored at −80 °C. These tissues were excess skin obtained from elective plastic surgeries that would have otherwise been discarded. Informed consent for the use of tissues in research was obtained by the physician. No identifiable patient health information was collected by BiopharmX or MGH. All experimental protocols at MGH were judged as not human research and approved under and exempt IRB ruling. All methods at MGH were carried out in accordance to safety guidelines approved by the Partners Institutional Biosafety committee. The frozen tissue specimens were thawed and portioned prior to experimentation. BPX-05 formulations containing 0% APIs (vehicle), 1% minocycline (MNC), 0.2% tazarotene (TAZ), and both 2% MNC and 0.2% TAZ (combination treatment), were applied onto the skin’s surface at 60 mg/cm^2^ with an O-rubber ring guide. Tissue samples receiving 60 mg/cm^2^ doses were incubated on a damp gauze pad at 32 °C for 24 hours. After incubation, the residual formulation on the skin surface was removed by gently wiping with an isopropyl alcohol (70%) pad, and the skin samples were trimmed with a scalpel. Subsequently, the trimmed tissues were embedded in optimal cutting temperature compound and frozen. Prior to measurement, the frozen tissue samples were cryo-cross sectioned along the plane perpendicular to the skin surface with a thickness of 30 µm using a cryostat (AVANTIK QS11, Springfield Township, NJ). The tissue sections were then mounted on microscope slides for FLIM measurements.

### Two-photon excitation fluorescence lifetime imaging microscopy (FLIM)

A confocal laser scanning microscope (Olympus FV1000, Center Valley, PA) with a 20x objective lens (0.75 NA, Olympus UPlanSAPO 20×, Center Valley, PA) was modified for use as a TPEF system. A widely tunable femtosecond laser (Spectra-Physics InSight DeepSee, Santa Clara, CA, 680 nm–1300 nm), was introduced into the microscope scanhead backport. The power was maintained below 30 mW at the sample for all imaging experiments to avoid any photodamage of tissue samples. Photobleaching of APIs was not observed under experimental conditions. A 680 nm shortpass filter in the epi collection path (Chroma E680SP-2P, Bellows Falls, VT) was used to reject any remaining 780 nm light from the excitation source. The emitted light was then passed through a bandpass filter centered at 525 nm with a 50 nm bandwidth (HQ525/50M, Chroma, Bellows Falls, VT) to filter emission light prior to detection with a photomultiplier tube (PMT; Hamamatsu H7422P-40, Hamamatsu City, Japan). To collect FLIM data, a commercial time-correlated single-photon counting (TCSPC) system (Becker & Hickl SPC150, Berlin, Germany) was used. Collected FLIM images were 256 × 256 pixels over a 635 × 635 μm field of view. Each image pixel contains a fluorescence decay trace histogram with 256 time bins across the 12.5 ns pulse repetition period of the laser source. A solution of fluorescein with a known lifetime of 4.05 ns at pH 9.0 was used to calibrate the instrument response function of the FLIM system. All FLIM images were acquired with a 90 second acquisition time by detecting the emitted fluorescence in the 500 to 550 nm spectral window. This integration time allowed for the collection of approximately 300 photons per pixel. In this manner, binning a 3 × 3 pixel region provided more than 2500 photons for analysis, which is considered adequate for a two or three-component lifetime fit^13^.

### Visualization of drug spatial distribution for single component topical drug

Previous studies have described and demonstrated the feasibility of FLIM along with phasor analysis for the sensitive visualization and quantification of a single component topical drug distribution^3,19^. Briefly, phasor analysis transforms all temporal fluorescence decay traces represented by pixels in a FLIM image into a pair of phasor coordinates (G, S) through a Fourier transform. G represents the real part of the Fourier transform and is plotted on the x-axis of phasor plots, while S represents the imaginary portion of the Fourier transform and is plotted on the y-axis of the phasor plot. This allows for a phasor plot to represent the lifetime properties of a fluorophore, appearing as clusters of fluorescence lifetime distributions arising from molecular microenvironmental heterogeneity. Fluorophores with sufficiently disparate fluorescence lifetimes will form spatially separated clusters within phasor space, allowing for the visualization of multiple compounds tagged with appropriate fluorophores. When two fluorophores contribute to a pixel’s temporal fluorescence decay trace, the resulting phasor coordinate is calculated through a linear combination of the phasor clusters representing the fluorophores. The proportion of the two fluorophores’ contributions to that pixel can then be computed by calculating the Mahalanobis distances between the sample’s phasor to the two phasor clusters of contributing fluorophores^20^.

In this study, to evaluate the uptake of each API (MNC or TAZ) within skin, three sets of experiments were carried out: (i) skin samples treated with only the vehicle were imaged to measure the endogenous fluorescence reference from the skin, (ii) each API was imaged to measure exogenous fluorescence references individually, and (iii) skin samples were treated with each topical drug (MNC or TAZ) individually to measure drug-skin interactions^3^. For single drug component applications, the imaging data is analyzed using the non-Euclidean phasor analysis approach. Then, the Mahalanobis distance between each pixel’s phasor coordinate to the endogenous and exogenous fluorescence reference clusters are calculated on a pixel-by-pixel basis to construct a local distribution map of drug within the skin. While the Mahalanobis distance-based approach is viable for single component drugs, it cannot be extended to multicomponent treatments due to ambiguities that arise from discerning cluster membership for a point between three or more reference phasor clusters.

### Visualization of drug spatial distribution for multicomponent topical drug

To quantitatively evaluate the local distribution of each component in the two-component MNC/TAZ topical formulation within the skin, phasor coordinates (G and S) were generated from four images: a test sample (a sample with a two-component API formulation applied), an endogenous reference (to visualize and serve as control for autofluorescence), an exogenous reference having only the API MNC, and an exogenous reference having only the API TAZ. The centers of the exogenous and endogenous phasor clusters were calculated by taking the mean G and S coordinates of each cluster. To account for the inherent noise in FLIM analysis, noise was purposely added to this calculation to account for measurement error and allow for better generalizability across different sets of exogenous, endogenous, and test samples. Each cluster’s variance was calculated by computing the distance from each point in the reference cluster to the center point of that cluster, and the variance of all clusters was summed into the total variance. Each cluster center point was then modified by adding 25% of its fraction of total variance to the x or y value of the center point. This moved the top-most reference cluster center point by 0.25 times the fraction of total variance from that cluster, times the original y coordinate, added to the original y coordinate:

1 $${{\rm{y}}}_{{\rm{n}}{\rm{e}}{\rm{w}}}={{\rm{y}}}_{{\rm{o}}{\rm{r}}{\rm{i}}{\rm{g}}{\rm{i}}{\rm{n}}{\rm{a}}{\rm{l}}}+({{\rm{y}}}_{{\rm{o}}{\rm{r}}{\rm{i}}{\rm{g}}{\rm{i}}{\rm{n}}{\rm{a}}{\rm{l}}}\,\ast \,0.25\,\ast \,{{\rm{v}}{\rm{a}}{\rm{r}}{\rm{i}}{\rm{a}}{\rm{n}}{\rm{c}}{\rm{e}}}_{{\rm{c}}{\rm{l}}{\rm{u}}{\rm{s}}{\rm{t}}{\rm{e}}{\rm{r}}})$$

The left-most cluster center was shifted left, and the right-most cluster center shifted right by a similar calculation applied to the x coordinate and subtracted/added to the original x coordinate respectively. This mathematical augmentation effectively expands the area enclosed by connecting the three cluster center points, and lessens the influence of higher-variance clusters on distance-based calculations by scaling clusters according to their individual variances. This procedure was inspired by oversampling procedures in machine learning, where datasets are stratified to balance proportions of different types of data that are put through analysis pipelines^21,22^. The oversampling allows for more cluster data points to have their drug contributions calculated with less bias towards the highest variance phasor cluster, in this case skin autofluorescence (AF) as the main contributor to fluorescence decay traces. It is known that skin AF is a combination of multiple fluorescent features of the skin, and is a larger, noisier cluster in the phasor space. Specifically, in the measurement conditions used for these experiments, the skin’s autofluorescence can be primarily attributed to flavins, FAD, keratins, elastins, and collagens, each of which have individually different lifetimes^23–25^. These contributions are effectively collapsed into a single AF cluster and are typically indistinguishable. Thus, the down-weighting of AF through noise addition allows for more focused analysis of APIs of interest.

**Figure 1:**
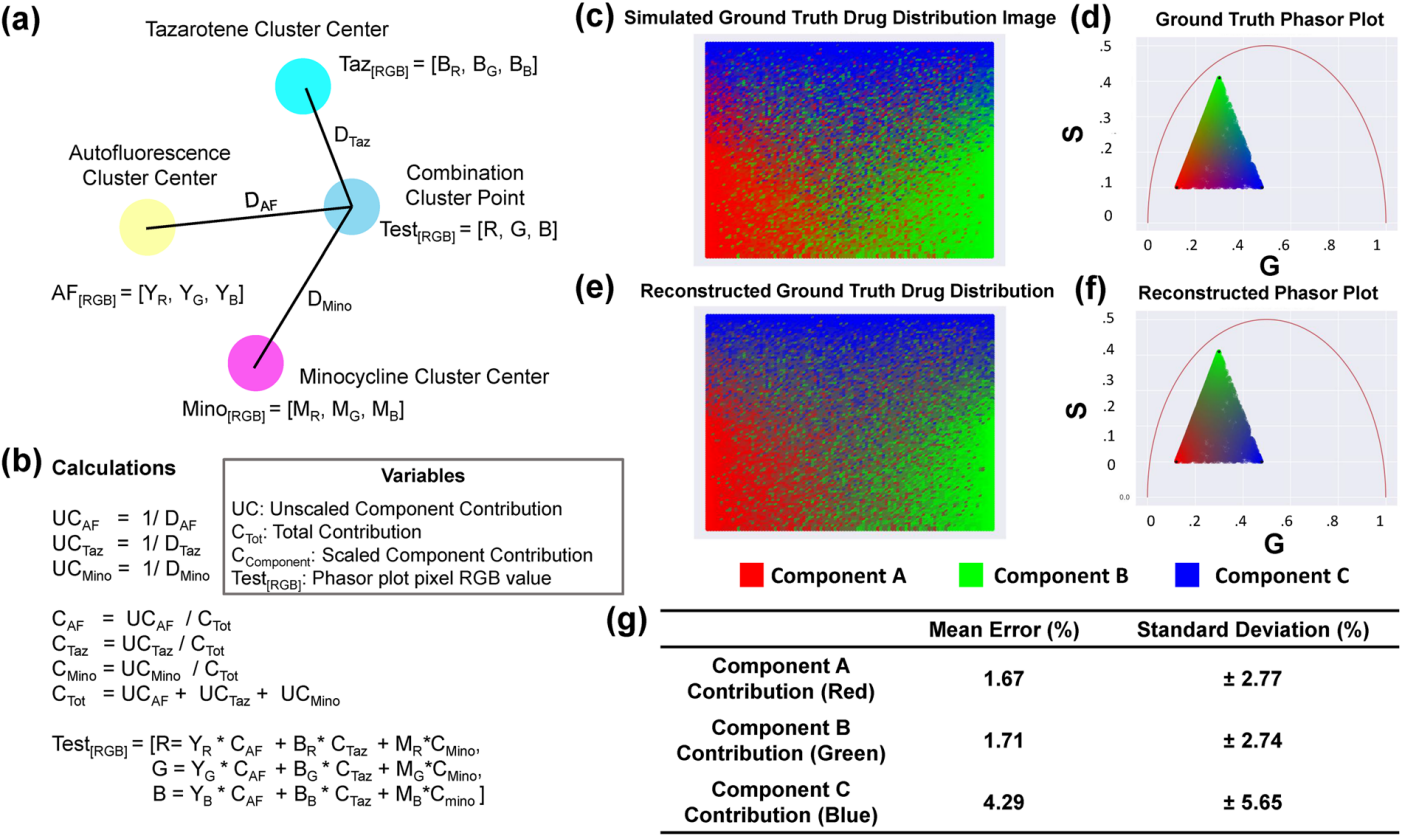
Distance-based method of assigning phasor plot pixel colors in a multicomponent fluorescence contribution analysis algorithm. (**a**) A sample setup of a distance calculation. The yellow, cyan, and magenta points represent the center of exogenous (autofluorescence) and two endogenous reference clusters (TAZ and MNC), respectively, and the blue point represents a point from the tissue sample FLIM image converted into phasor space. Each cluster center point is assigned a color value and is of variable distance from the phasor point of test sample. (**b**) The variables and calculations used to determine the RGB value of the phasor point from the test sample. Using the inverse of the measured distance between reference clusters and the phasor point of test sample allows for proximal reference cluster(s) to have a higher impact on the pixel RGB color and thus the composition of the test phasor is RGB color-coded at that point. (**c**) Simulated ground truth tissue map of drug distribution. (**d**) Phasor plot calculated based on contributions of different components in the ground truth tissue map. (**e**) Reconstructed tissue drug distribution using the multicomponent phasor analysis algorithm. (**f**) Reconstructed phasor plot coming from contributions calculated in the multicomponent phasor analysis algorithm. (**g**) Metrics assessing accuracy of the ground truth reconstruction on a per-point, per-component basis in the simulated tissue map.

Following center point noise augmentation, normalized contributions to individual phasor points from exogenous and endogenous clusters were calculated by computing the distances D_Exo1_, D_Exo2_, D_Endo_ from a point P to the one endogenous and two exogenous cluster centers. Contributions C_Exo1_, C_Exo2_, C_Endo_ were defined as 1/D _Exo1_, 1/D _Exo2_, and 1/D _Endo_ respectively. These contributions were then normalized by C_tot_ = C_Exo1_ + C_Exo2_ + C_Endo_ (Fig. 1a,b). The endogenous and exogenous components were assigned distinct colors, set by the user. Yellow was selected to represent AF, cyan to represent TAZ, and magenta to represent MNC; these specific colors were chosen to avoid potential issues visualizing RGB patterns for colorblind individuals. For each pixel in the MNC-TAZ combination treatment phasor plot, a new color was assigned by scaling each color value from each cluster center point by the normalized contribution of that center point to the pixel. This color mapped phasor plot was converted back to Cartesian space while maintaining its color assignment to visualize the endogenous or exogenous contributions present throughout the tissue sample.

### Simulating data to benchmark multicomponent algorithm performance

To validate the phasor distance-based multicomponent visualization algorithm, simulated data was generated and analyzed to benchmark algorithm performance. The goal was to create a simulated map of drug distribution in tissue, where (x,y) coordinate values in the images were assigned phasor values; analysis of this simulated data allows for the assessment of the multicomponent visualization algorithm’s accuracy. For this unit test, a synthetic ground truth map of tissue autofluorescence and a two-component drug distribution image was created. First, individual maps of the three different components were created then added together to generate a map showing the contributions of the multiple components in the tissue. In the simulated drug and autofluorescence distribution tissue map, peak component concentrations were maximally separated in the image, with the highest contributions of two components being in opposite corners along the x-axis of the image, and the center of the third component centered along the horizontal line at the maximum y-value in the image. The contribution of each component to a point in the simulated tissue map was decreased along a gradient, such that the center of the simulated tissue image had nearly equal contributions from the three components. Symmetric linear gradients were used for the two components centered in the synthetic tissue image corners, while a radial gradient was used for the third component centered in the top middle of the image. Poisson noise was added to the simulated compound contributions prior to assigning phasor coordinates to each pixel of the simulated tissue image, corresponding to each component’s simulated fluorescence lifetime and phasor cluster center. Phasor coordinates were assigned by choosing three points as the variance-adjusted centers of each component’s phasor cluster, corresponding to the point in the simulated tissue drug distribution map with pure contribution from one component. After assigning phasor coordinates, the multicomponent visualization algorithm was applied in order to optimally recreate the original simulated tissue image. To assess error between ground truth and recreated simulated tissue images, mean squared percent error was measured on a per-component basis (Fig. 1).

## Results

### Characterization of minocycline, tazarotene, and skin’s autofluorescence using two-photon excitation fluorescence spectroscopy and fluorescence lifetime imaging microscopy (FLIM)

In order to understand the native fluorescence parameters of both MNC and TAZ, fluorescence excitation-emission (EEM) spectra were recorded from each drug dissolved in their topical delivery formulation (BPX-05). These EEMs revealed that the two APIs have different, but overlapping, maximum excitation peaks (MNC at 480 nm and TAZ at 410 nm) while showing similar maximum emission spectra (MNC at 510 nm and TAZ at 490 nm, Fig. S1 in the Supplementary Information). By analyzing the maximum two-photon excitation fluorescence signal from each API, we experimentally determined that 780 nm was the optimal two-photon excitation wavelength for both compounds. Two-photon fluorescence emission spectra of the two APIs (MNC and TAZ) and skin’s autofluorescence were then obtained at an excitation wavelength of 780 nm. The two APIs showed strikingly similar spectral emission profiles that both overlapped with that of skin autofluorescence (peak at *ca*. 520 nm, Fig. S2 in the Supplementary Information). When the sample solutions were dried via exposure to ambient atmosphere for more than three hours, MNC’s dried form displayed a red-shifted fluorescence emission (centered at *ca*. 560 nm) while TAZ’s dried form showed a blue-shifted fluorescence emission (centered at *ca*. 485 nm) (Fig. S2 in the Supplementary Information). Although the drying process provided potential leverage to differentiate MNC from TAZ, both of APIs’ emission spectra still significantly overlapped with skin’s AF emission, thus limiting the isolation of individual compound signal from AF. Follow up two-photon excitation FLIM experiments allowed for the extraction of lifetime parameters that suggested the ability uniquely identify the imaged molecular species.

**Figure 2:**
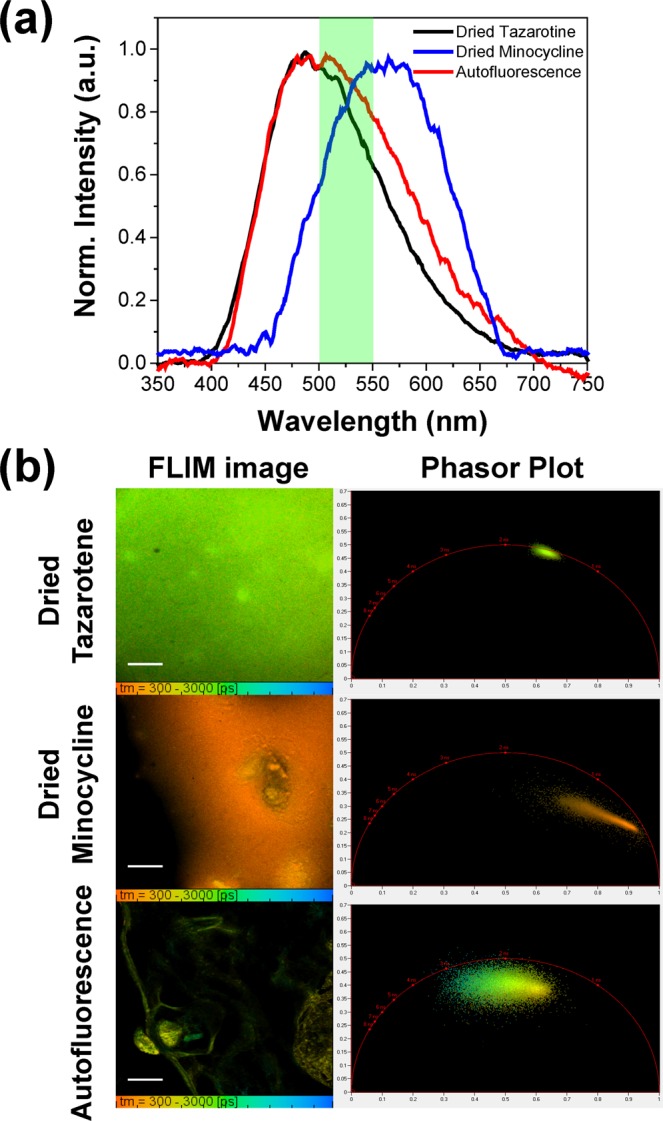
Characterization of fluorescence from dried form minocycline, tazarotene, or skin’s autofluorescence by (**a**) two-photon excitation fluorescence (TPEF) spectra and (**b**) fluorescence lifetime imaging microscopy (FLIM). The spectra were obtained with a 10-s acquisition time while the 780 nm laser (two-photon excitation) continuously scanned the sample. The FLIM images were acquired from 500–550 nm channel with 780 nm excitation. The FLIM images were color-coded according to the calculated fluorescence lifetime by fitting the fluorescence decay trace of each pixel from FLIM image with a double-exponential decay function on a pixel-by-pixel basis. The phasor plots corresponding to the FLIM images were generated using SPCImage software (Becker & Hickl GmbH). The scale bar is 100 μm.

To investigate the feasibility of FLIM for differentiating the fluorescence signals of MNC, TAZ and AF, FLIM images of dried tissue, dried MNC, and dried TAZ were obtained from the 500–550 nm emission channel. This channel sufficiently captured MNC, TAZ and AF emissions. As shown in Fig. 2b, the fluorescence lifetime of each sample was computed through time-correlated single photon counting (TCSPC) analysis, where fluorescence decay traces are fit with a double exponential decay function. The choice of a double exponential function here was a practical one, designed to help visually elucidate potential changes in fluorescence lifetime in the image data. From the TCSPC analysis, it was found that the MNC dried form had a shorter fluorescence lifetime (0.4–0.5 ns) than the TAZ dried form (1.4 ns) and AF (1.5–2.5 ns). By employing phasor analysis, each phasor cluster corresponding to MNC, TAZ, and AF, can be seen to be spatially distinct in phasor space, potentially allowing for the isolation of each topical drug’s signal from the skin’s autofluorescence (Fig. 2). This result indicates that the phasor approach can provide an efficient means to selectively isolate the fluorescence contribution of each drug from the overwhelming and overrepresented autofluorescence signal.

### Exploration of topical drug uptake within *ex vivo* facial skin using FLIM

**Figure 3:**
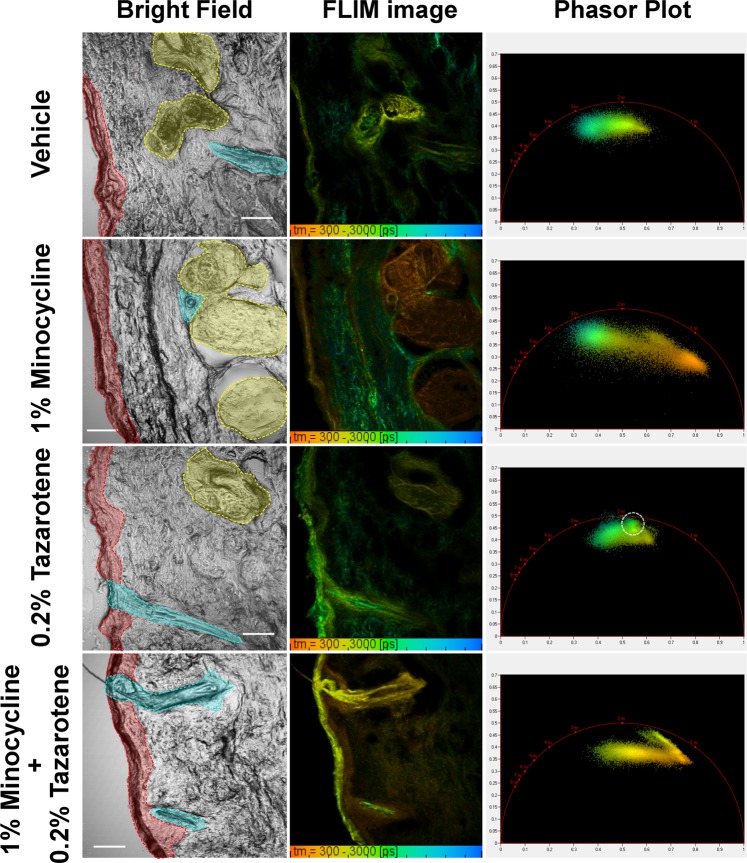
Visualization of uptake of the active pharmaceutical ingredient (MNC and/or TAZ) in the topical drug treated *ex vivo* facial skin using FLIM along with time-correlated single photon counting (TCSPC) analysis and phasor analysis. The facial skin was treated with 60 mg/cm^2^ BPX-05 containing 0% APIs (vehicle), 1% MNC, 0.2% TAZ, and both of 1% MNC and 0.2% TAZ for 24 hours. The anatomical features of skin were color-annotated in bright field images: epidermis (red), hair follicle (cyan), and sebaceous gland (yellow). The FLIM images were acquired from the 500–550 nm channel with 780 nm excitation and 90-s acquisition time. The TCSPC-FLIM images were generated by fitting a double or triple exponential decay functions to each pixel’s decay trace. The phasor plots of FLIM images were generated by SPCImage software (Becker & Hickl GmbH). The white circle in the phasor plot of the TAZ treated sample indicates the phasor cluster region associated with TAZ uptake. The scale bar is 100 μm.

To verify the applicability of the FLIM-based visualization method for analyzing topical drug uptake, *ex vivo* facial skin tissue sample was treated with topical drug (60 mg/cm^2^ BPX-05 containing either 1% minocycline, 0.2% tazarotene, or both 1% MNC and 0.2% TAZ) for 24 hours. The resulting samples were cryo-cross sectioned and mounted on the glass slide. As mentioned in the previous section, the drying process allows these APIs to have distinct fluorescence emissions as well as fluorescence lifetimes. To exploit this unique property and distinguish fluorescence contributions of individual compounds in the MNC + TAZ treated skin sample, the cross-sectioned tissue samples were dried in ambient atmospheric condition for more than 3 hours. This period allowed MNC and TAZ to be fully dried within anatomical skin features such as the epidermis, hair follicles, and sebaceous glands. To generate a skin autofluorescence reference dataset, skin samples were treated with the BPX-05 vehicle only and imaged without APIs added. The FLIM image of this vehicle-treated sample showed the expected fluorescence lifetime of tissue autofluorescence (1.5–2.5 ns, Fig. 3, row 1) within the epidermis as well as the sebaceous gland, consistently spread throughout the sample. In the phasor plot, AF lifetimes form a cluster towards the middle of the universal semicircle. In contrast, skin treated with 1% MNC treated demonstrated a cluster with a shorter lifetime (*ca*. 0.4–0.5 ns, Fig. 3, row 2) that was primarily localized in the topmost layer of the epidermis. While this layer of skin was in direct contact with the applied minocycline API, MNC was also detected within its target, the sebaceous gland. The MNC phasor cluster appeared more elongated and distinctly off-center relative to the AF cluster, indicative of increased MNC contribution to the image pixel. This unique elongated phasor cluster demonstrated the MNC’s uptake in the epidermis as well as the sebaceous gland.

The TAZ treated sample showed a longer lifetime contribution (*ca*. 1.4 ns, Fig. 3, row 3) at the epidermis, hair follicle, and sebaceous gland. In the phasor plot, a distinct cluster region (indicated with white circle in the phasor plot in Fig. 3, row 3) appears shifted towards the reference phasor cluster of TAZ, indicating TAZ uptake into the epidermis and deep layers of skin along the hair follicle, particularly via the infundibulum. While the majority of TAZ’s individual cluster is obscured by AF, this specific region and the overall shift of the cluster of points towards the TAZ cluster and is indicative of TAZ uptake. In the combination (1% MNC and 0.2% TAZ) treated sample (bottom row of Fig. 3), a lifetime of 0.8–1 ns was observed at the epidermis, which was thought to be due to colocalization of MNC and TAZ. Due to MNC having a shorter fluorescence lifetime (0.4 ns) and TAZ having a longer fluorescence lifetime (1.4 ns), the resulting image is hypothesized to yield the mean fluorescence lifetime (0.8–1 ns). In contrast, the lifetime of *ca*. 0.4 ns within the hair follicle, particularly the infundibulum, suggests that MNC uptake is relatively higher at this location than TAZ uptake. This interpretation was found to match the results of the phasor analysis, which demonstrated distinct elongated elliptical clusters spanning the space between two reference phasor clusters of MNC and TAZ dried form. The spreading out of the phasor cluster indicates the colocalization of MNC and TAZ over a range of different drug uptake distributions.

### Quantification of local distribution of single component API within facial skin using non-Euclidean phasor analysis

**Figure 4:**
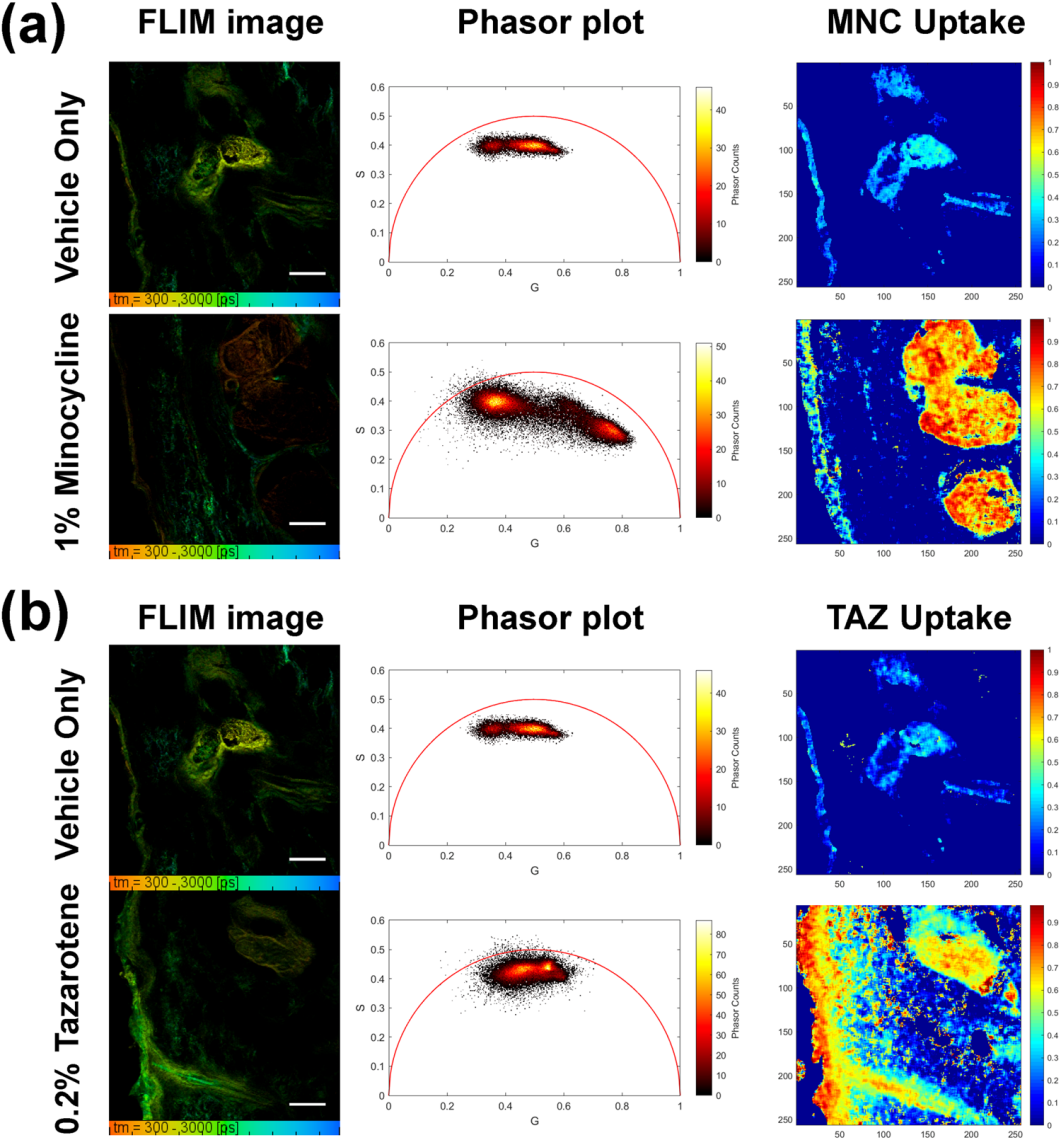
Quantitative visualization of individual API uptake (MNC or TAZ) in the single component topical drug using the non-Euclidean phasor analysis algorithm. TSCPC-FLIM images of the *ex vivo* facial skin samples treated with BPX-05 containing no APIs (vehicle), and either 1% MNC or 0.2% TAZ were obtained from 500–550 nm with 780 nm two-photon excitation and 90 s acquisition time. The (**a**) MNC or (**b**) TAZ uptake images were computed using the non-Euclidean phasor analysis algorithm with two fluorescence references including one endogenous (skin’s autofluorescence) and one exogenous (MNC or TAZ dried form, respectively). The scale bar is 100 μm.

While the lifetime data suggests uptake patterns attributable to each API, these results must be studied and quantified to understand the uptake of each individual compound in skin. In order to quantitatively visualize uptake of individually-applied topical drugs, the data from single topical drug treated samples were processed using non-Euclidean phasor analysis^3^. As described in the methods, the two fluorescence contributions to each pixel in the FLIM image can be computed by calculating pixel-wise Mahalanobis distances. This calculation was carried out between each phasor pixel of sample’s FLIM image and both compound reference phasor clusters. The vehicle only treated skin sample served as a reference of endogenous fluorescence and the measured topical drug dried form (MNC or TAZ) served the exogenous fluorescence reference. Using non-Euclidean phasor analysis, topical drug uptake maps were generated with values between zero and unity, corresponding to the drug’s fluorescence contribution in each pixel of the FLIM image. As shown in Fig. 4a, only the MNC treated sample showed the elongated elliptical phasor cluster that lied on along the line between the two reference clusters in the phasor plot. Furthermore, the generated MNC uptake map showed high uptake in the sebaceous gland as well as the epidermis.

On the other hand, the TAZ treated sample showed a unique cluster shifted towards TAZ’s reference cluster (Fig. 4b). More importantly, the TAZ uptake map clearly and quantitatively showed the local distribution of TAZ uptake in the epidermis followed by the infundibulum, which could not be clearly differentiated in the TCSPC images above. It should be noted that while the vehicle only treated sample did not contain any MNC and TAZ, the calculated images here show what seem to be weak false positive drug uptake of both MNC and TAZ in the sebaceous gland and epidermis. These artifacts are attributed to the wide heterogeneity of autofluorescence lifetimes and non-uniformity of endogenous fluorophores within the skin, and are a minor contribution to the observed signal.

These results reinforce the applicability of the non-Euclidean phasor analysis approach for the quantification of single-agent topical APIs, and clearly show the uptake pattern of each individual API. This approach, however, cannot be readily applied to cases where there are more than one API. Doing so would require a computationally intensive quadratic component in the phasor analysis to isolate each of three components’ contributions (AF, MNC, and TAZ) in each sample’s pixel. Several previous studies suggested multicomponent phasor analysis by calculating the distance of the sample’s phasor to three different references’ phasor clusters^16^. However, when applied to the data in this study, these suggested phasor approaches provided less distinguishability and generated high false-positives due to the heterogeneity of the references’ fluorescence lifetimes (broad phasor cluster) and the proximity of the references’ phasor clusters, motivating the need for a new approach (Fig. S3 in the Supplementary Information).

### Quantification of drug distribution within facial skin using multicomponent phasor analysis

In order to improve multicomponent phasor analysis, a contribution analysis algorithm was developed that can account for multiple exogenous reference clusters in the phasor space. One of the central challenges for isolation of each API fluorescence contribution in this study is the proximity of the three references’ phasor clusters and location of the combination phasor cluster. To address these key challenges, noise was mathematically introduced in a post-processing step to each of the references’ phasor clusters that allows for additional augmented “spatial” separation between phasor clusters in phasor space. This transformed dataset enabled a wider range of combination treatment phasor points to be measured. This approach was validated by creating synthesized image data that simulates a varying tissue autofluorescence background with a two-component drug mixture (Fig. 1c–f). This simulated data set up scenarios where individual pixels in the image could have major contributions from one, two, or all three fluorophores. This synthetic “ground truth” image data was then processed using the multicomponent phasor analysis, and the constructed output was compared to the original. For pixels that contained one or two primary fluorescence lifetime contributions, the mean error was low, at approximately 2%. Pixels that contained contributions up to three simulated fluorophores have a slightly higher error rate of 5%, as a greater number of component contributions to pixels have more introduced Poisson noise in the simulation (Fig. 1g). However, the error rate for this simulated three-component analysis is overall quite low and is well within the acceptable margins of error.

**Figure 5:**
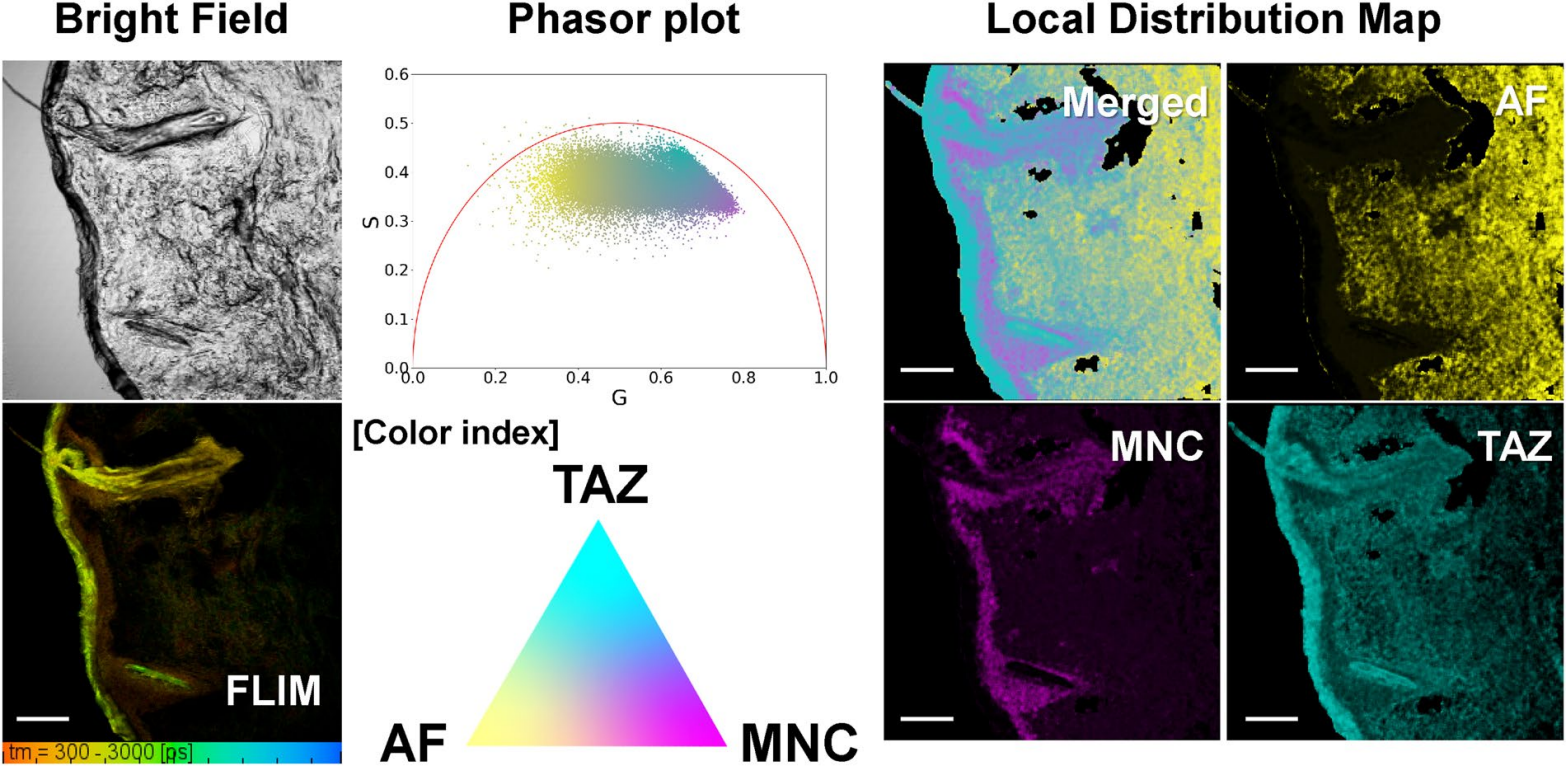
Quantitative visualization of double APIs (MNC and TAZ) in the *ex vivo* facial skin sample treated with BPX-05 containing both 1% MNC and 0.2% TAZ using the multicomponent fluorescence contribution analysis algorithm. The FLIM image was obtained from 500–550 nm channel with 780 nm two-photon excitation. The TCSPC-FLIM image was generated by fitting the triple-exponential decay function to each fluorescence decay trace from a pixel of FLIM image. The three contributions of fluorescence references at each pixel’s phasor in the phasor plot and the individual local distributions of three fluorescence references were quantitatively visualized with the color-index (yellow for AF, cyan for TAZ, and magenta for MNC, respectively) by using the multicomponent fluorescence contribution analysis algorithm. The scale bar is 100 μm.

As mentioned in the methods, the mathematical addition of noise caused the y coordinate of the topmost cluster center to become vertically shifted, and the left and right most clusters to become horizontally shifted (Fig. S4 in Supplementary Information). To evaluate the feasibility of this approach for visualizing multicomponent topical drug local distribution, FLIM images from both of MNC and TAZ treated sample were reanalyzed. As depicted in Fig. 5, each distinct phasor cluster center point is assigned a color – yellow for AF, cyan for TAZ, and magenta for MNC. Thus, each API is uniquely identified in the treatment phasor plot and local contribution image, while being distinct from AF. This analysis uses the distance of a test point to a reference cluster center to measure the contribution probability at the test point as a function of reference clusters in the phasor space. Each pixel in the treatment sample phasor cluster is assigned a color value (between 0 and 255 in each channel) through inversely weighing the distance from that pixel to each noise-added exogenous and endogenous cluster center. This means, for example, that a treatment data point twice as close to the TAZ reference cluster as it is to either MNC or AF would have twice as much cyan value in its color than it would have yellow and magenta. This allows for the creation of smooth colormaps depicting the transition of exogenous and endogenous cluster contribution to each point in the treatment sample. These colormaps are initially generated in the phasor space before being translated to the treated tissue sample coordinate system that was imaged via FLIM. This approach to quantifying multicomponent FLIM images allows for more complete compensation of the heterogeneity of biomedical samples.

As shown in Fig. 5, the multicomponent analysis generates a complete 1:1 mapping between the input treatment tissue image and visualized compounds in the tissue. Across different test samples with combination drug treatment applied, the consistent trend involves TAZ being delivered and retained in outer layers of facial skin, followed by high likelihood regions of MNC uptake slightly deeper in the tissue. However, there is a stark contrast between where MNC uptake stops and where AF becomes most prominent in the tissue sample - corresponding to where the APIs likely failed to diffuse at high levels. TAZ likelihood reached a maximum value of 0.991 in the outer tissue region, MNC reached a maximum in the region between TAZ and AF at a value of 0.517 and AF reached a maximum in the inner facial tissue region with a value of 0.994 (Fig. S5 in the Supplementary Information).

## Discussion

Quantitative evaluation of drug delivery at intended target sites is crucial for assessing drug functionality. When topical drugs have fluorescence properties, FLIM can be a powerful tool for extracting PK profiles within the skin by directly visualizing the local distribution of topical drug uptake. The key challenge in this method is that skin has a highly heterogeneous composition, including multiple endogenous fluorophores^26^. Moreover, the range of microenvironment in skin tissue can alter the fluorescence lifetimes of APIs taking up into the skin. This alteration of APIs’ fluorescence lifetime in the skin makes it even more difficult to quantify the local contribution of APIs uptake using TCSPC-FLIM analysis because of its increased complexity. Applying phasor analysis for FLIM images can directly visualize this alteration and heterogeneity of APIs’ fluorescence lifetimes in the phasor space, allowing for further quantitative analysis of local API distribution within the skin.

Here we have reported an optical PK imaging approach that can visualize multiple topical drugs within skin tissue specimens and assess local drug distribution in skin. We previously demonstrated the feasibility of a non-Euclidean phasor approach for FLIM analysis to selectively quantify and visualize the uptake of a topical minocycline formulation within facial skin. In this study, this method was extended to obtain the local distribution of individual APIs within a multi-compound topical drug applied to skin. Previous phasor-based approaches to analyzing FLIM data were limited by the inability to disambiguate overlapping contributions between endogenous and exogenous clusters in phasor space, placing the goal of examining multiple drugs out of reach.

In the previous approach^3,19^, cluster overlap between exogenous and endogenous species in the phasor space resulted in less accurate quantification and visualization of compound interactions at a point. However, considering the distance of a point in the phasor space to the centers of reference clusters now allows for a less biased calculation of compound contribution to a point in the treatment tissue sample. An important feature of the multicomponent drug distribution mapping is the addition of noise to the calculation of the center points of reference clusters. This allows for more combination treatment phasor points to be included in the final calculation of drug presence in the tissue. The noise added is proportional to the variance of the phasor cluster, causing center coordinates from larger clusters to have greater noise added than compact exogenous clusters. This acts to mitigate the oversized impact of larger clusters and more accurately represents the behavior of compounds in skin samples while controlling for potential measurement error. Furthermore, this approach allows for more generalizability across different tissue samples tested with the same imaging parameters. This noise addition method was initially created to control for autofluorescence, which is an agglomeration of non-fluorescently labeled artifacts in the skin. Without incorporating clusters variance into contribution calculations, the phasor cluster representing autofluorescence would appear overrepresented and falsely overshadow the drug compounds in the skin. This, in turn, causes inaccuracies with quantification of compound presence in the tissue. As shown with synthesized data, the error rate of this phasor transformation-based analysis is low, and the accuracy of the ground truth reconstruction is trustworthy. In the future, modifying the ‘direction’ that noise is added to be along the slope of the line of best fit of a cluster might result in slightly higher quantification accuracy. However, in this scenario, given that the noise-added center was still well within each cluster distribution, adding purely horizontal or vertical modifications to the cluster center does not invalidate the distance calculations.

As discussed, MNC contribution probabilities appeared lower than TAZ and AF probabilities, likely due to the treatment phasor cluster being shifted towards AF and TAZ references. This does not mean that MNC uptake is low, but based on local distribution maps in Fig. 5, means that MNC phasor pixels seem to colocalize with TAZ pixels, thus lowering the maximum possible contribution probability. The probability density function showing the distribution of contribution probabilities shows a high density of middle-range probability values, indicative of colocalization (Fig. S5 in Supplementary Information). Additionally, this could be due to MNC’s readily separable phasor cluster compared to the overlapping clusters of TAZ and AF. Nonetheless, high confidence regions of MNC presence in tissue are clearly visible, meaning that the quantification method can account for colocalization by determining AF and TAZ contributions to be lower relative to MNC contributions.

In the FLIM imaging condition for Fig. 5, TAZ showed stronger fluorescence signal than MNC. Since TCSPC-FLIM systems record the time between sample excitation laser pulse and the first arrival of the emitted single photon at detector, MNC uptake in the Fig. 5 may have been underestimated because of its weaker fluorescence signal. Future research incorporating optimized imaging conditions for both MNC and TAZ might provide more accurate cluster center measurements and better drug permeation mappings. Furthermore, analysis of TAZ and MNC optimized images may result in higher maximum contribution probabilities for MNC to sample phasor cluster points.

Importantly, this study demonstrated that an evidently high quantity of APIs exists in the pilosebaceous unit of the tissue, which is the desired spatial skin target for the two APIs (Fig. S6 in the Supplementary Information). The maps generated through phasor distance calculations in this approach are relative local maps, but do not provide absolute drug concentrations or PK parameters. These maps allow for more robust qualitative analysis than previously available, which is a positive step towards absolute quantification. Incorporating TAZ and MNC optimized imaging protocols could provide a means for developing an algorithm for quantifying absolute parameters for multicomponent drugs. In the future, testing different topical formulations on tissue samples would allow us to assess the generalizability of the multicomponent phasor analysis across different fluorescence lifetimes, phasor cluster geometries, and allow for PK parameter extraction. Upon future method optimization, integrating the analysis technique presented here with commercially available real-time or portable FLIM systems would allow for rapid assessment of drug uptake properties in human clinical studies^12,27,28^. This approach may be important in the development and formulation of new topical drugs, new topical drug mixtures, as well as a tool in determining topical generic drug bioequivalence.

## Supplementary information

Supplementary information.

## References

[1] Gregoriou S, Kritsotaki E, Katoulis A, Rigopoulos D (2014). Use of tazarotene foam for the treatment of acne vulgaris. Clin. Cosmet. Investig. Dermatol..

[2] Gollnick HPM, Krautheim A (2003). Topical Treatment in Acne: Current Status and Future Aspects. Dermatology.

[3] Jeong S (2018). Visualization of drug distribution of a topical minocycline gel in human facial skin. Biomed. Opt. Express.

[4] Smith K, Leyden JJ (2005). Safety of doxycycline and minocycline: A systematic review. Clin. Ther..

[5] Goulden V, Glass D, Cunliffe WJ (1996). Safety of long-term high-dose minocycline in the treatment of acne. Br. J. Dermatol..

[6] Okada N (1993). Characterization of pigmented granules in minocycline-induced cutaneous pigmentation: observations using fluorescence microscopy and high-performance liquid chromatography. Br. J. Dermatol..

[7] Leyden JJ (2003). A review of the use of combination therapies for the treatment of acne vulgaris. J. Am. Acad. Dermatol..

[8] Thielitz A, Sidou F, Gollnick H (2007). Control of microcomedone formation throughout a maintenance treatment with adapalene gel, 0.1%. J. Eur. Acad. Dermatol. Venereol..

[9] Thielitz A, Gollnick H (2008). Topical Retinoids in Acne Vulgaris. Am. J. Clin. Dermatol..

[10] Sorensen IS (2017). Combination of MALDI-MSI and cassette dosing for evaluation of drug distribution in human skin explant. Anal. Bioanal. Chem..

[11] Yamada M (2018). Using elongated microparticles to enhance tailorable nanoemulsion delivery in excised human skin and volunteers. J. Control. Release.

[12] Alex A (2018). *In situ* biodistribution and residency of a topical anti-inflammatory using fluorescence lifetime imaging microscopy. Br. J. Dermatol..

[13] Raufast V, Mavon A (2006). Transfollicular delivery of linoleic acid in human scalp skin: permeation study and microautoradiographic analysis. Int. J. Cosmet. Sci..

[14] Fereidouni F, Bader AN, Colonna A, Gerritsen HC (2014). Phasor analysis of multiphoton spectral images distinguishes autofluorescence components of *in vivo* human skin. J. Biophotonics.

[15] Stringari C (2011). Phasor approach to fluorescence lifetime microscopy distinguishes different metabolic states of germ cells in a live tissue. Proc. Natl. Acad. Sci..

[16] Ranjit S, Malacrida L, Jameson DM, Gratton E (2018). Fit-free analysis of fluorescence lifetime imaging data using the phasor approach. Nat. Protoc..

[17] Digman MA, Caiolfa VR, Zamai M, Gratton E (2008). The phasor approach to fluorescence lifetime imaging analysis. Biophys. J..

[18] Colyer R (2012). Phasor imaging with a widefield photon-counting detector. J. Biomed. Opt..

[19] Osseiran S (2017). Non-Euclidean phasor analysis for quantification of oxidative stress in *ex vivo* human skin exposed to sun filters using fluorescence lifetime imaging microscopy. J. Biomed. Opt..

[20] Mahalanobis PC (1936). On the Generalized Distance in Statistics. Proc. Natl. Inst. Sci..

[21] Chawla NV, Bowyer KW, Hall LO, Kegelmeyer WP (2002). SMOTE: synthetic minority over-sampling technique. J. Artif. Intell. Res..

[22] Lemaître G, Nogueira F, Aridas CK (2017). Imbalanced-learn: A python toolbox to tackle the curse of imbalanced datasets in machine learning. J. Mach. Learn. Res..

[23] Skala MC (2007). *In vivo* multiphoton microscopy of NADH and FAD redox states, fluorescence lifetimes, and cellular morphology in precancerous epithelia. Proc. Natl. Acad. Sci..

[24] Huang S, Heikal AA, Webb WW (2002). Two-Photon Fluorescence Spectroscopy and Microscopy of NAD(P)H and Flavoprotein. Biophys. J..

[25] Shirshin EA (2017). Two-photon autofluorescence lifetime imaging of human skin papillary dermis *in vivo*: assessment of blood capillaries and structural proteins localization. Sci. Rep..

[26] Hermsmeier M (2018). Characterization of human cutaneous tissue autofluorescence: implications in topical drug delivery studies with fluorescence microscopy. Biomed. Opt. Express.

[27] Poulon F (2018). Real-time Brain Tumor imaging with endogenous fluorophores: a diagnosis proof-of-concept study on fresh human samples. Sci. Rep..

[28] Koenig K (2012). Hybrid multiphoton multimodal tomography of *in vivo* human skin. IntraVital.

