# Supplementary material for: Time-resolved fluorescence microscopy with phasor analysis for visualizing multicomponent topical drug distribution within human skin
Source: Sci Rep. 2020 Mar 24;10:5360. doi: 10.1038/s41598-020-62406-z (PMC7093415; doi:10.1038/s41598-020-62406-z)
Supplement: Supplementary file 1 — Supplementary information. [file 41598_2020_62406_MOESM1_ESM.pdf]

[Supplementary Information]

Time-resolved fluorescence microscopy with phasor analysis for visualizing multicomponent topical drug distribution within human skin

Sinyoung Jeong,[a] Daniel A. Greenfield,[a,b] Maiko Hermsmeier,[c] Akira Yamamoto,[c] Xin Chen,[c] Kin F. Chan,[c] Conor L. Evans[a,b,*]

[a] Wellman Center for Photomedicine, Massachusetts General Hospital, Harvard Medical School, Boston, MA 02114, USA
[b] Harvard Biophysics Program, Medical School Campus, Boston, MA 02115, USA
[c] BioPharmX, Inc., 115 Nicholson Ln, San Jose, CA 95134, USA

Contents

Figure S1. Fluorescence excitation-emission matrix (EEM) of minocycline and tazarotene dissolved in the topical drug formulation (BPX-05).

Figure S2. Two-photon excited fluorescence emission of autofluorescence, minocycline and tazarotene dissolved in the topical drug formulation as well as those dried forms.

Figure S3. Quantitative visualization of double APIs (MNC and TAZ) in the *ex vivo* facial skin sample treated with BPX-05 containing both 1% MNC and 0.2% TAZ using previously proposed Euclidian phasor analysis.

Figure S4. Demonstration of the effect of adjusting the computed cluster centers.

Figure S5. Distribution of contribution probabilities for reference clusters.

Figure S6. Quantitative visualization of double APIs (MNC and TAZ) in the *ex vivo* facial skin sample treated with BPX-05 containing both 1% MNC and 0.2% TAZ using the multicomponent fluorescence contribution analysis algorithm.

Reference

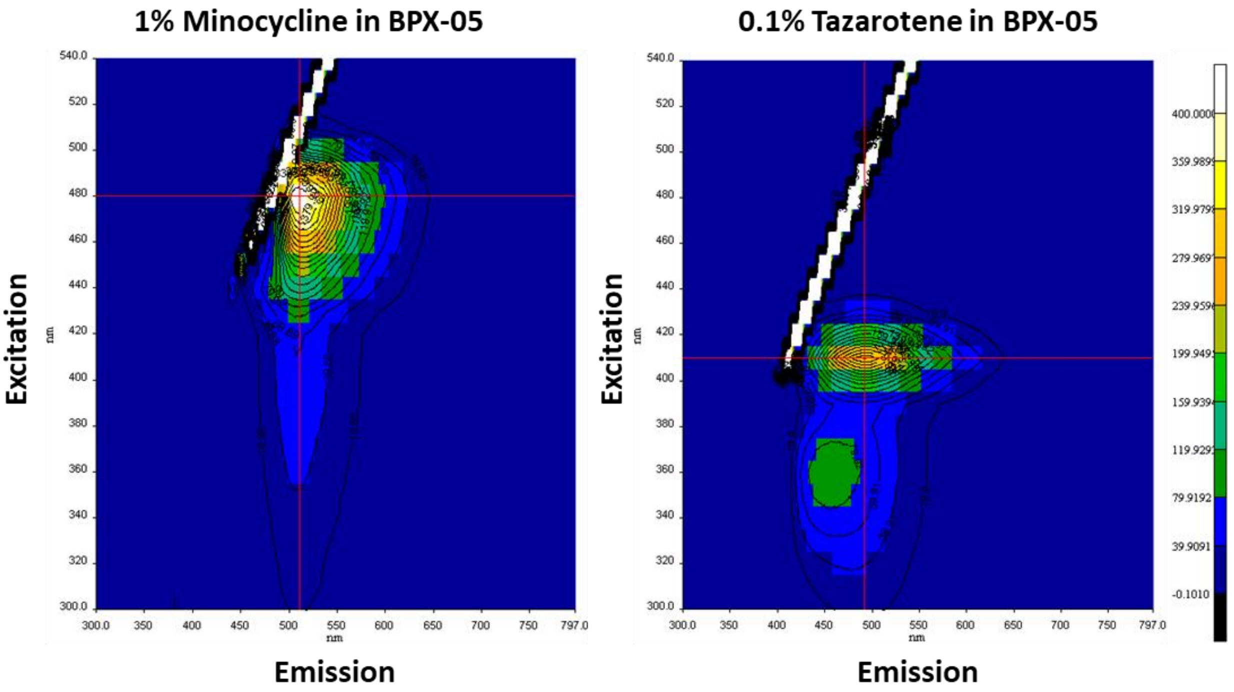

Figure S1. Fluorescence excitation-emission matrix (EEM) of minocycline and tazarotene dissolved in the topical drug formulation (BPX-05).

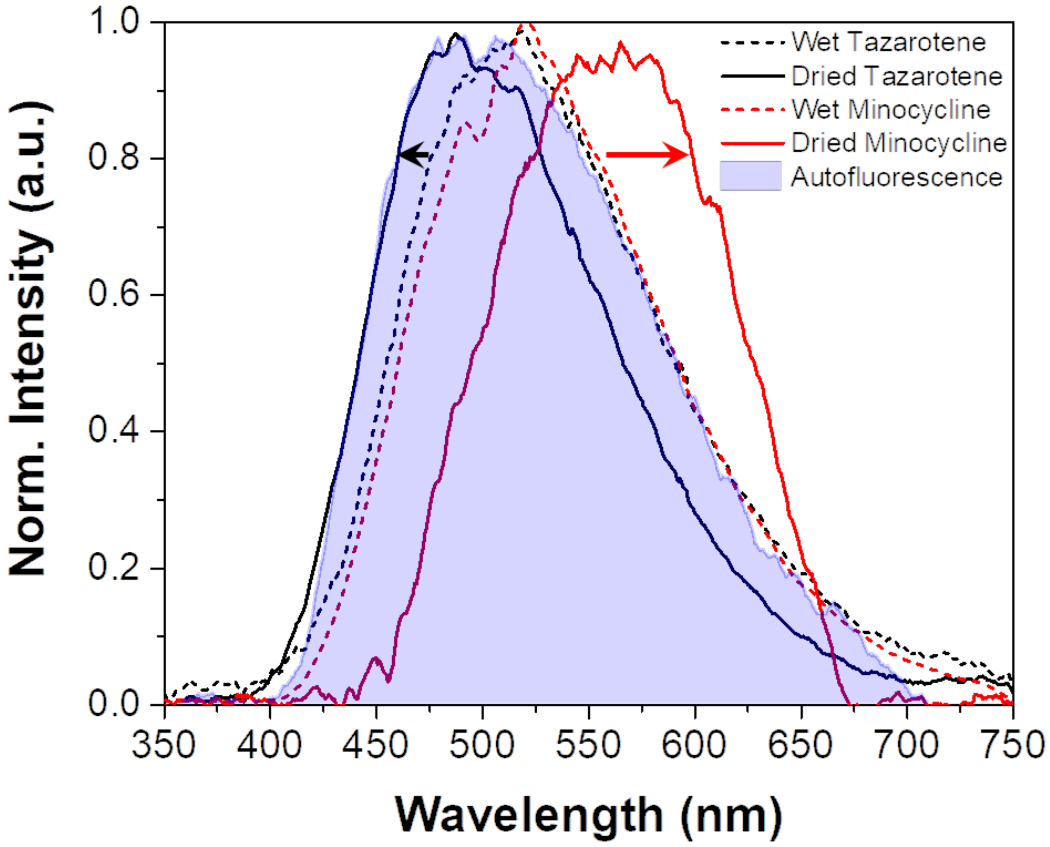

Figure S2. Two-photon excited fluorescence emission of autofluorescence, minocycline and tazarotene dissolved in the topical drug formulation as well as those dried forms. The spectra were obtained with 780 nm two-photon excitation and 10-s acquisition time by a spectrometer (Ocean Optics QE65000, Largo, FL) coupled an optical fiber (Thorlabs M25L02, 200 µm silica core, NA 0.22, Newton, NJ) in conjunction with a collimation lens (Thorlabs F810SMA-635, NA 0.25, Newton, NJ).

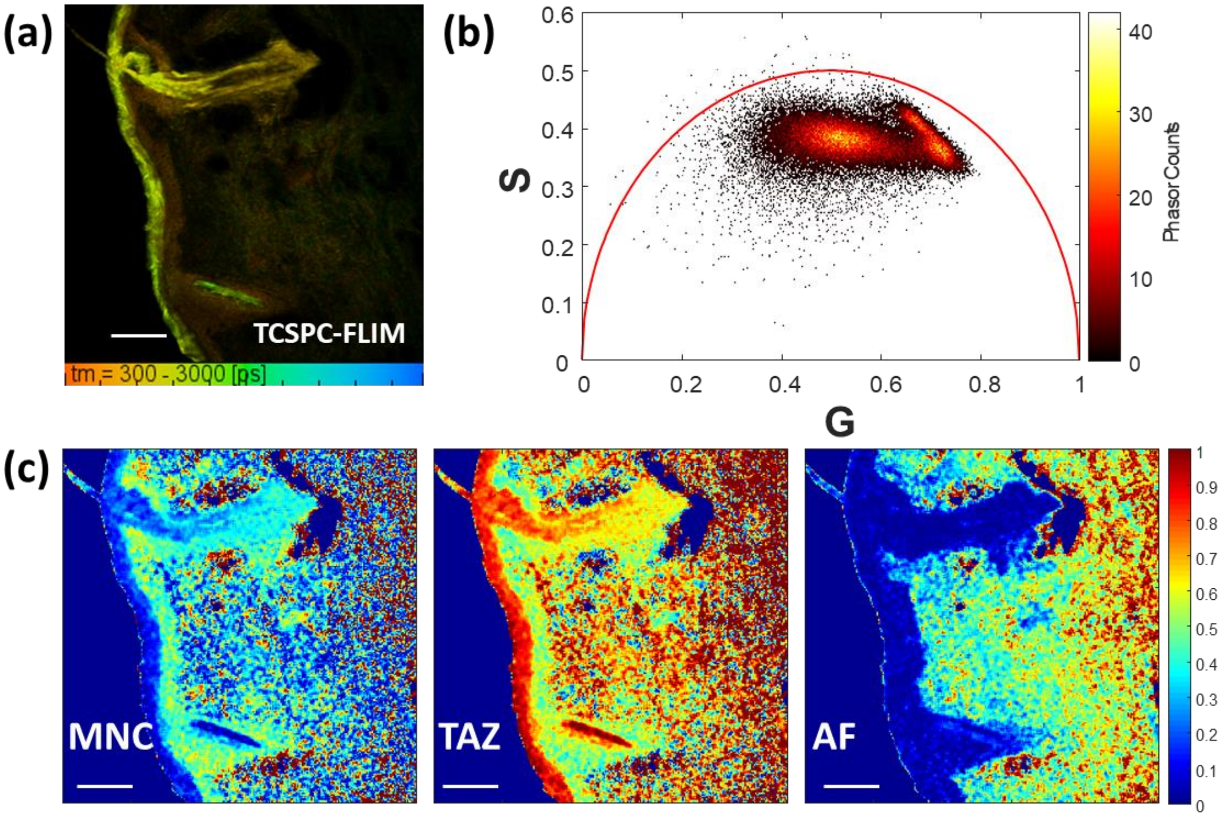

Figure S3. Quantitative visualization of double APIs (minocycline, MNC and tazarotene, TAZ) in the ex vivo facial skin sample treated with BPX-05 containing both 1% MNC and 0.2% TAZ using previously proposed Euclidian phasor analysis. (a) The TCSPC-FLIM image was generated by fitting the triple-exponential decay function to each fluorescence decay trace from a pixel of FLIM image. The fluorescence lifetime was computed by fitting the fluorescence decay traces with triple-exponential decay function. (b) The phasor plot associated with FLIM image and (c) the individual local distributions of three fluorescence references (MNC, TAZ, and autofluorescence, AF) were generated by Euclidean phasor analysis algorithm.[1] It was noticed that high false positive contribution both of MNC and TAZ (close to 1.0 contribution) at the dermis area. The scale bar is 100 μm.

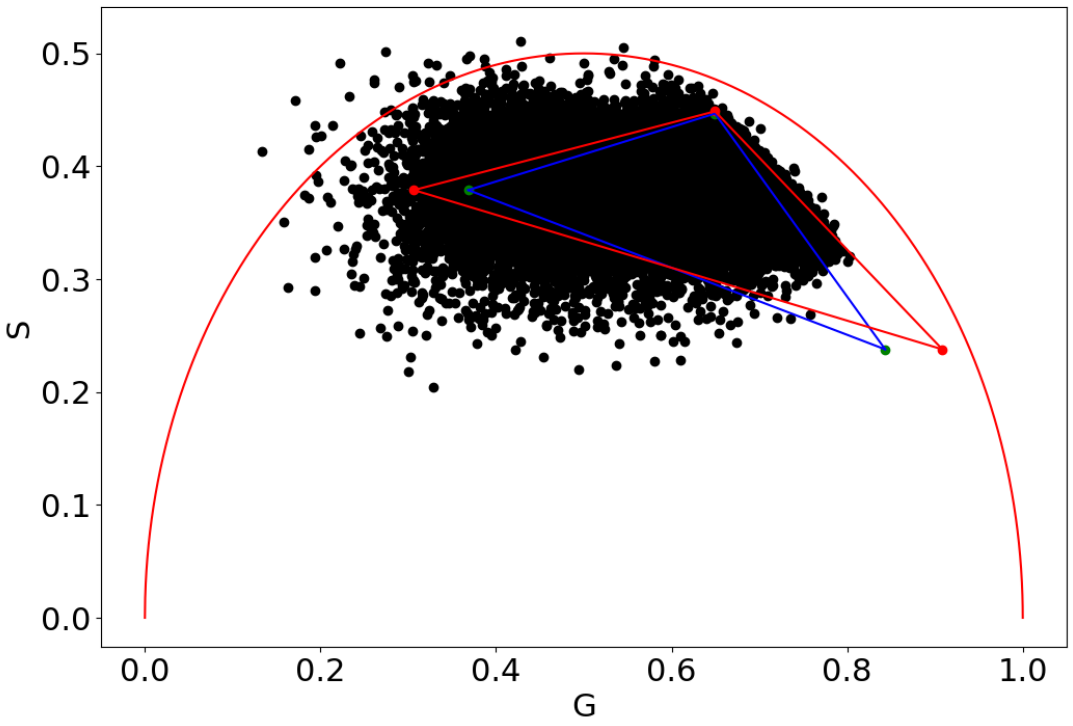

Figure S4. Demonstration of the effect of adjusting the computed cluster centers. In blue is the triangle created by connecting the three original cluster centers, and in red is the triangle that connects the variance-adjusted cluster centers. Low variance clusters, such as the one represented in as the top vertex of the triangle, have smaller adjustments added to their coordinates prior to contribution calculation than larger variance clusters do (rightmost vertex).

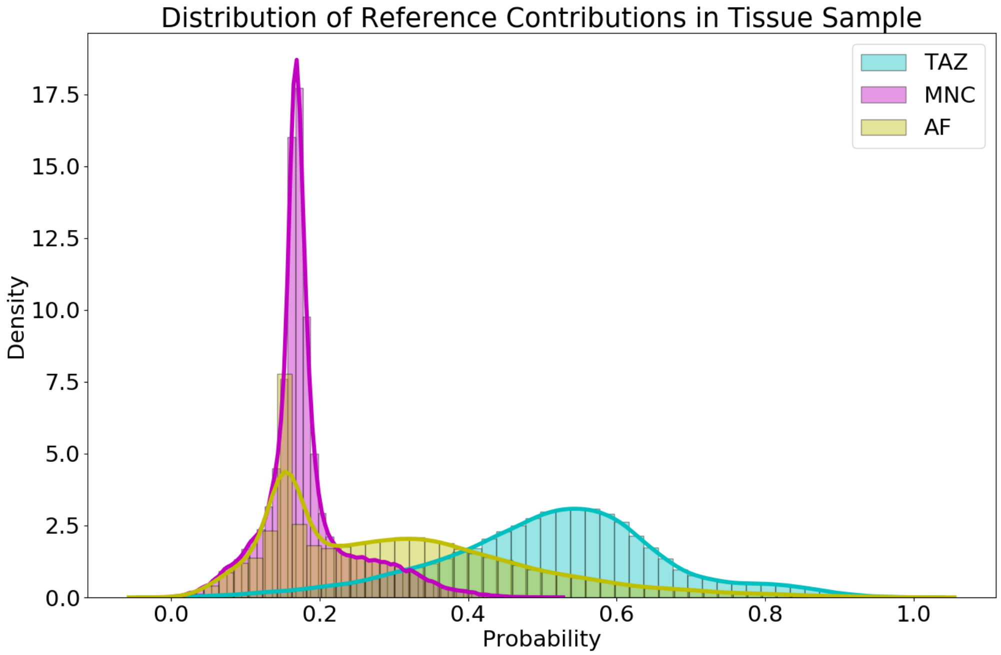

Figure S5. Distribution of contribution probabilities for reference clusters. The probability axis represents the calculated contribution probability based on inversely weighing distance, and the density axis represents the number of points with that calculated probability. Colocalization of MNC and AF in the tissue result in similar probability distributions, while TAZ is visibly distinct.

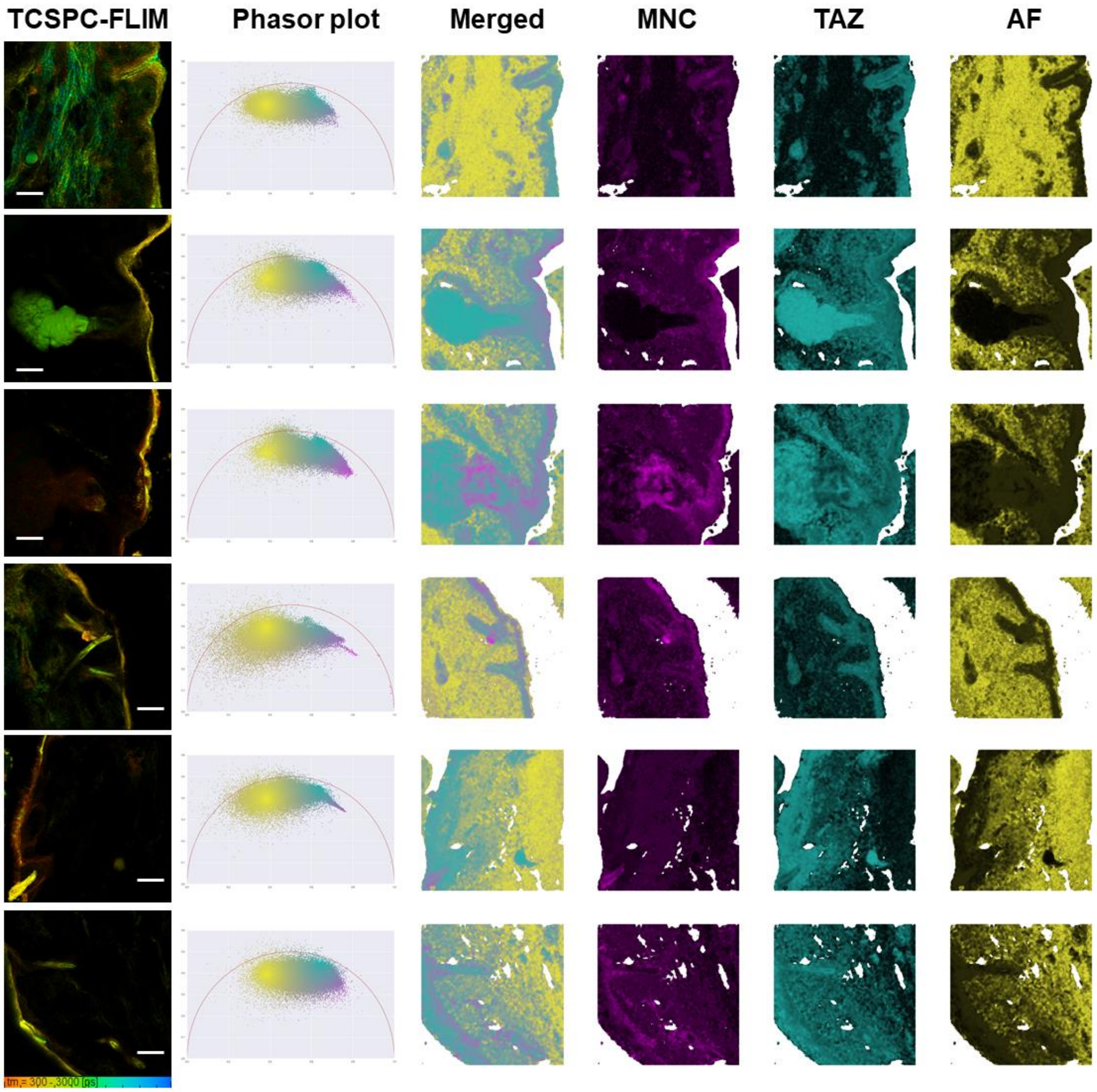

Figure S6. Quantitative visualization of double APIs (MNC and TAZ) in the *ex vivo* facial skin sample treated with BPX-05 containing both 1% MNC and 0.2% TAZ using the multicomponent fluorescence contribution analysis algorithm. The FLIM image was obtained from 500-550 nm channel with 780 nm two-photon excitation. The TCSPC-FLIM image was generated by fitting the triple-exponential decay function to each fluorescence decay trace from a pixel of FLIM image. The three contributions of fluorescence references at each pixel's phasor in the phasor plot and the individual local distributions of three fluorescence references were quantitatively visualized with the color-index (yellow for AF, cyan for TAZ, and magenta for MNC, respectively) by using the multicomponent fluorescence contribution analysis algorithm. The scale bar is 100 μm.

Reference

(1) Ranjit, S.; Malacrida, L.; Jameson, D. M.; Gratton, E. *Nat. Protoc.* **2018**, *13*, 1979-2004.
